# Intact fibroblast growth factor 23 and fragments in plasma from Gambian children

**DOI:** 10.1007/s00198-012-2029-3

**Published:** 2012-05-31

**Authors:** V. Braithwaite, S. F. A. Bruggraber, A. Prentice

**Affiliations:** 1 MRC Human Nutrition Research, Elsie Widdowson Laboratory, Cambridge, CB1 9NL United Kingdom; 2 MRC Keneba, Keneba, West Kiang, The Gambia

**Keywords:** FGF23, Gambian rickets, Western blotting

## Abstract

**Summary:**

Fibroblast growth factor 23 (FGF23) is grossly elevated in Gambian children with rickets and, at a lower prevalence, in those without bone deformities. We used western blotting to mimic the detection capabilities of the C-terminal FGF23 enzyme-linked immunosorbent assay (ELISA). Only intact FGF23 hormone was present in Gambian plasma samples from children with and without rickets.

**Introduction:**

Elevated circulating FGF23 concentrations have been detected in plasma samples from Gambian children using the C-terminal Immutopics ELISA. The Immutopics ELISA detects both the intact FGF23 hormone and the C-terminal fragment. The aim of this study was to determine whether the elevated FGF23 concentrations as detected by the ELISA were predominantly due to a high proportion of intact FGF23 hormone and/or C-terminal FGF23 fragments.

**Methods:**

Stored, frozen plasma samples from previous studies of Gambian children with known concentrations of FGF23 as determined by C-terminal Immutopics ELISA assay, were selected for western blotting analysis: from children with rickets-like bone deformities (*n* = 4) and local controls (*n* = 4), with elevated >900 RU/ml (*n* = 2) and normal <30 RU/ml (*n* = 2; from each group). The anti-FGF23 polyclonal antibody that recognizes the C-terminal of FGF23 (as used in the Immutopics kit) was used as the primary antibody and the anti-IgG polyclonal antibody conjugated to horseradish peroxidase (HRP) was used as the secondary antibody.

**Results:**

Firstly, C-terminal FGF23 fragments, although detectable in standards from the Immutopics ELISA kit, were not in the Gambian plasma samples. Secondly, there was no difference in the size of FGF23 molecules present in plasma from children with rickets-like bone deformities and children from the local community.

**Conclusions:**

Western blotting has provided evidence that elevated FGF23 concentrations, as determined by the C-terminal Immutopics ELISA, measured in Gambian children with and without rickets-like bone deformities was not caused by an increased proportion of circulating inactive C-terminal fragments.

## Introduction

Fibroblast growth factor 23 (FGF23) is a phosphate-regulating hormone produced primarily by osteocytes [1]. FGF23 expression is predominantly regulated by plasma phosphate (P) [2] and 1,25-dihydroxyvitamin D (1,25-(OH)_2_D) [3]. The principal target organ of FGF23 is the kidney where it causes the internalization of sodium–phosphate cotransporters in renal tubular cells and the suppression of 1α-hydroxylase activity [4], thus decreasing plasma P by increasing urinary phosphate excretion and down-regulating 1,25-(OH)_2_D concentrations, respectively.

**Fig. 1:**
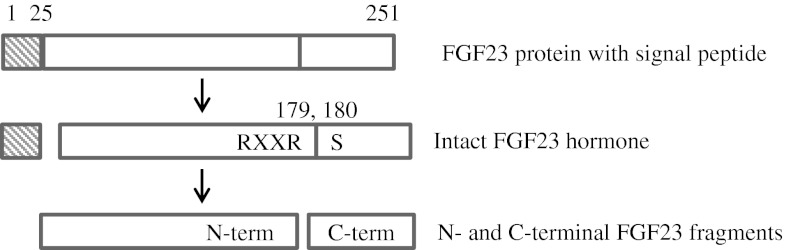
Schematic of the FGF23 protein starting with the full FGF23 product (251 amino acids), the signal peptide (24 amino acids) is then cleaved off to produce the intact FGF23 hormone which is considered biologically active. Proteolytic cleavage then occurs at the end of the RXXR motif between R^179^ and S^180^ to produce the biologically inactive N- and C-terminal fragments. Both the intact hormone and the C-terminal fragments are recognized by the C-terminal Immutopics ELISA assay [8]

The FGF23 gene encodes the 251 amino acid FGF23 peptide, which includes a signal peptide (SP) of 24 amino acids. Prior to secretion the SP is cleaved to form the intact FGF23 protein. The intact FGF23 protein contains the arginine–X–X–arginine (RXXR) motif which is a protease recognition site [5]. When proteolytically cleaved between Arg^179^ and Ser^180^ the intact FGF23 (~32 kDa) forms an N- and C-terminal (~12 kDa) fragment (Fig. 1). It is thought that only the intact FGF23 protein is biologically functional and that the cleavage step forming the N- and C-terminal fragments renders the protein inactive [6].

There are currently two commercially available enzyme-linked immunosorbent assays (ELISA) for measurement of FGF23 concentration, namely the Kainos Intact FGF23 ELISA (Kainos Laboratories, Inc., Tokyo, Japan) and the Immutopics C-terminal FGF23 ELISA (Immutopics, Inc., CA, USA). The Intact ELISA uses two antibodies that recognize the N-terminal and C-terminal regions and therefore only recognizes the full, intact FGF23 hormone prior to proteolytic cleavage. However, the two antibodies used in the C-terminal ELISA detect epitopes within the C-terminal region and therefore recognizes both the intact hormone and the C-terminal fragment.

Grossly elevated FGF23 concentrations have been detected in a large percentage of Gambian children with rickets-like bone deformities using the C-terminal ELISA [7]. These were associated with elevated 1,25-(OH)_2_D and, for patients with active rickets, hypophosphatemia [7, 8]. Chronic calcium deficiency has been proposed as a likely etiological factor [7]. Additionally, albeit at a lower prevalence, elevated FGF23 concentrations have also been detected in a small percentage of local reference children with no signs of bone deformities [9]. The aim of the study was to determine whether C-terminal FGF23 fragments were present in Gambian plasma samples and therefore detected using the Immutopics ELISA and if this was different in plasma from children with and without rickets-like bone deformities. Western blot analysis was used with the anti-FGF23 polyclonal antibody that recognizes the C-terminal of FGF23 (as used in the Immutopics kit) as the primary antibody and the anti-IgG polyclonal antibody conjugated to HRP as the secondary antibody. This method was intended to replicate the detection capabilities of the Immutopics ELISA and to thus identify what FGF23 protein/fragments were being detected.

## Methods

### Subject population

**Fig. 2:**
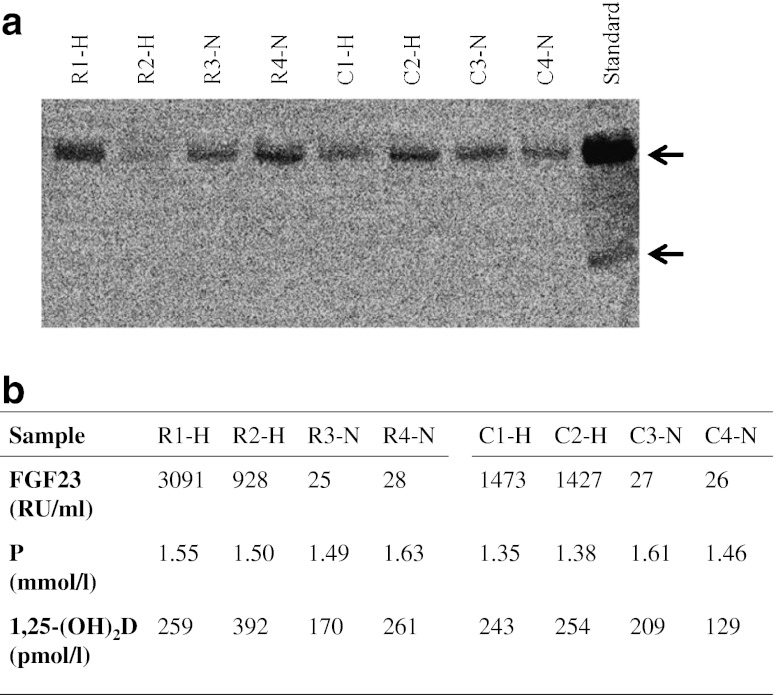
Western blot **a** of plasma samples from four rickets children (R1-R4) and four local community children with **b** previously measured elevated (H) and normal (N) FGF23 concentrations, plasma phosphate (P) and 1,25-dihydroxyvitamin D (1,25-(OH)_2_D) and a standard from the Immutopics ELISA kit. The arrows indicate the intact FGF23 protein and the C-terminal fragment. There is no evidence of C-terminal protein in the plasma samples but there is in the standard **a**

Fasted EDTA plasma samples (*n* = 8) from an etiological study of rickets in Gambian children were selected from stored frozen samples collected from children with a history of rickets-like bone deformities and from the local community [7–9] (Fig. 2b) in whom plasma FGF23 (C-terminal ELISA; Immutopics, USA), phosphate (colorimetric; Koni Analyser 20i, Finland) and 1,25-(OH)_2_D (radioimmunoassay; IDS, UK) concentrations had been previously determined. According to the manufacturer’s instruction, FGF23 concentration at 25–125 RU/ml is regarded as the normal range. For the western blot analysis, we selected four children (two with and two without a history of rickets-like bone deformity) with a very high FGF23 (>900 RU/ml) and four children (two with and two without a history of rickets-like bone deformity) with FGF23 concentration within the normal range. None of the subjects had active disease or hypophosphatemia at the time the blood sample was taken [8, 9]. Ethical approval was obtained from The Gambian Government/MRC Laboratories Joint Ethics Committee to conduct further studies on FGF23 using these stored samples.

### SDS-PAGE and western blot analysis

Plasma samples and standards from the ELISA kit (50 μl) were filtered using 50 kDa Amicon ultra centrifugal filters (Milipore, UK) to remove proteins with a molecular weight greater than 50 kDa. A 5 μl aliquot of plasma filtrate was mixed with 1 μl NuPAGE® reducing agent, 2.5 μl NuPAGE® sample buffer and 1.5 μl of water according to manufacturer’s instructions (Invitrogen Ltd, Paisley, UK). Any bubbles were removed and the samples were denatured by heating for 15 min at 75 °C and then placing on ice for 10 min. The samples were then loaded onto NuPage® 4–12 % Bis-Tris gels (Invitrogen Ltd, Paisley, UK) and were separated at 200 V for 25 min. The proteins were then transferred onto a nitrocellulose membrane (Invitrogen Ltd, Paisley, UK) using the Xcell blot II Module (Invitrogen Ltd, UK) for 1 h at 30 V using NuPAGE® transfer buffer (Invitrogen Ltd, Paisley, UK) according to manufacturer’s instructions. Membranes were incubated in blocking solution (5 % dry fat-free milk powder in phosphate buffered saline (PBS)–Tween solution (PBS with 0.1 % Tween-20; Sigma-Aldrich Company Ltd, Dorset, UK) for 2 h at room temperature. Membranes were then incubated in the primary antibody, anti-FGF23 polyclonal antibody that recognizes the C-terminal of FGF23, diluted 1:1,000 with the blocking solution for 1 h at room temperature. Membranes were then washed with PBS-Tween and then incubated with the secondary antibody, donkey polyclonal antibody to Goat IgG conjugated to HRP (Abcam, Cambridge, UK), diluted 1:2,000 in the blocking solution for 30 min at room temperature. Membranes were then washed with PBS-Tween and incubated with the substrate (Amersham ECL Plus Western Blotting Detection System; GE Healthcare Life Sciences, UK) for a short time before being exposed to a CCD camera (Alpha Innotech Imager) to capture the resulting chemiluminescent signal.

### Protein staining

After SDS-PAGE, the gels were stained using the Colloidal Blue Staining Kit (Novex®, Invitrogen Ltd, Paisley, UK) and dried using DryEase® Mini-Gel Drying System (Invitrogen Ltd, Paisley, UK) according to manufacturer’s instructions.

## Results

Using the anti-FGF23 polyclonal antibody that recognizes the C-terminal of FGF23, two bands were detected in the standard material from the ELISA kit namely, at approximately ~32 kDa and at a lower molecular weight ~12 kDa suggestive of the full-length intact FGF23 and C-terminal fragment, respectively. This indicated the western blot method is capable of detecting both intact and C-terminal FGF23 fragments. The Gambian plasma samples were then used in the same method and only one band was detected, at ~32 kDa, namely the full-length intact FGF23 hormone. There was no evidence of the presence of non-intact FGF23 hormone in the plasma samples and there was no difference in proteins detected in the samples from children with rickets-like bone deformities (R1–R4) and from local community children (C1–C4; Fig. 2a). Furthermore, protein staining indicated a ~32 kDa band in both the plasma samples and the standard material and a lower molecular weight band (~12 kDa) in the standard material but there were no low molecular weight proteins in the plasma samples.

## Discussion

We have previously reported the presence of elevated FGF23 concentrations in Gambian children with a history of rickets-like bone deformities [7, 8] as determined by the C-terminal Immutopics ELISA assay. Albeit at a lesser prevalence, we have also reported elevated FGF23 concentrations in children from the local community [8]. It has been suggested that these measurements could be a reflection of the inactive C-terminal fragments detected by the Immutopics ELISA and therefore not a true reflection of the concentrations of biologically functional intact FGF23 hormone. In order to explore this eventuality we used the same antibody as the C-terminal Immutopics ELISA kit in a western blot to determine which protein fragments were being detected by the ELISA. This confirmed detectable fragments in the standard material but not in the Gambian samples. This suggests that the high FGF23 concentrations, as measured by the C-terminal Immutopics ELISA in Gambian children with and without bone deformities, are a reflection of circulating intact FGF23 protein rather than high levels of cleaved product. Furthermore, protein staining indicated that there were no proteins of low molecular weight in the plasma samples suggesting the absence of any type FGF23 fragments, not only C-terminal fragments. Limitations of this study include the small number of plasma samples available for the analysis. In conclusion, a difference in proportion of cleaved FGF23 hormone does not explain the presence of high FGF23 in Gambian children with rickets-like bone deformities and in children from the local community [8].
